# Diagnosing sputum/smear-negative pulmonary tuberculosis: Does fibre-optic bronchoscopy play a significant role?

**DOI:** 10.4103/0970-2113.63607

**Published:** 2010

**Authors:** Arshad Altaf Bachh, Rahul Gupta, Inaamul Haq, Hanumant Ganapati Varudkar

**Affiliations:** *Department of Pulmonary Medicine, Mamata Medical College and Hospital, Khammam, Andhra Pradesh, India*; 1 *Community Medicine, Mamata Medical College and Hospital, Khammam, Andhra Pradesh, India*

**Keywords:** Bronchial washings, fibreoptic bronchoscopy, pulmonary tuberculosis, sputum smear negative

## Abstract

**Background:**

Diagnosis of sputum/smear-negative pulmonary tuberculosis patients can be both challenging and time consuming with many patients being put on empirical anti-tubercular treatment. Fibreoptic bronchoscopy may provide a confirmative and early diagnosis in such patients.

**Aims:**

To assess the role of fibreoptic bronchoscopy in the diagnosis of sputum /smear-negative pulmonary tuberculosis.

**Materials and Methods:**

The study was conducted on 75 suspected sputum / smear-negative pulmonary tuberculosis cases attending Pulmonary Medicine Department of Mamata Medical College and Hospital, Khammam, AP. Fibreoptic bronchoscopy was performed; culture of sputum and bronchial washings for *Mycobacterium tuberculosis* was done by BACTEC method.

**Results:**

A final diagnosis of sputum /smear-negative pulmonary tuberculosis was made in 60 patients. Bronchial washings smear for acid-fast bacilli (AFB) was positive in 21 patients while culture of bronchial washings was positive in 39 patients. In 29 patients, smear or culture of bronchial washing alone contributed to the final diagnosis. Total yield of bronchoscopy in diagnosis of sputum smear negative pulmonary tuberculosis was 83.33% (50/60); bronchoscopy was the only diagnostic method in 66% cases (40/60) with bronchial washings being the only diagnostic method in 48.33%. Bronchial washings smear for AFB and histopathological evidence of caseating granuloma made immediate diagnosis possible in 48.33% (29/60) patients.

**Conclusion:**

Our study suggests that fibreoptic bronchoscopy can provide excellent material for diagnosis of suspected cases of Pulmonary Tuberculosis in whom smears of expectorated sputum do not reveal mycobacteria.

## INTRODUCTION

The 1990 World Health Organization (WHO) report on the Global Burden of Disease ranked tuberculosis as the seventh most morbidity-causing disease in the world, and expected it to continue in the same position up to 2020.[1] Someone somewhere contracts tuberculosis every four seconds and one of them dies every 10 seconds.[2,3] In 2006, about 1.4 million cases of tuberculosis were registered for treatment in India; 28.7% of them were new smear negative cases.[4]

The initial diagnostic approach to suspected cases of pulmonary tuberculosis is to demonstrate *Mycobacterium tuberculosis* in stained smears of expectorated sputum. In most of the tuberculosis centers, even after meticulous search, the bacteriological positive yield from sputum is around 16 to 50% and large portion remain negative in spite of clinical profile and radiological lesions being consistent with diagnosis of pulmonary tuberculosis.[5] Early diagnosis of pulmonary tuberculosis prevents progression of disease, morbidity, spread of disease and permanent damage by fibrosis. Culture of sputum for acid fast bacilli (AFB) takes long time and a reliable serological test is not yet available. In such a situation bronchoscopy has been tried for rapid diagnosis of tuberculosis in smear negative cases. Fibreoptic bronchoscopy with bronchial washing analysis for AFB including culture for *Mycobacterium tuberculosis* has significant role to establish the diagnosis when extensive search for AFB in expectorated sputum has repeatedly failed, when sputum expectoration is absent or sputum induction has failed.

The present study aims to assess the role of fibreoptic bronchoscopy in the diagnosis of sputum/smear-negative pulmonary tuberculosis.

## MATERIALS AND METHODS

The present study, approved by the institutional ethics committee, was conducted in the Department of Pulmonary Medicine, Mamata Medical College and General Hospital, Khammam over a period from November 2005 to November 2007.

Clinically suspected cases of pulmonary tuberculosis, aged 16-75 years, with three sputum smears negative for AFB and a chest radiograph suggestive of pulmonary tuberculosis were included in the study after obtaining an informed consent. Patients with bleeding diathesis, history of myocardial infarction or arrhythmia, extra-pulmonary tuberculosis, history of anti-tubercular treatment (ATT) for more than one month, and those with severe dyspnoea were excluded from the study. HIV-positive and non-cooperative patients were also excluded.

A detailed history, clinical examination, and routine investigations were carried out on suspected cases of tuberculosis. Three sputum samples (spot, morning and spot) were tested for presence of AFB in the smear. In patients with suspected smear negative pulmonary tuberculosis, a sputum sample was sent for sputum culture (BACTEC) and the patients were taken up for bronchoscopy. Prior to the procedure an informed written consent was obtained from the patient. The procedure was carried out electively with the patient nil orally for four to six hours. Patients were pre-medicated 30-45 minutes prior to bronchoscopy with 0.6 mg atropine and nebulization was done with two per cent xylocaine via ultrasonic nebulizer. Bronchoscopy was carried out under local anesthesia. Olympus BF type E2 bronchoscope was used.

Bronchial washing was performed by instilling 0.9% isotonic saline at room temperature through the internal channel of the fibreoptic bronchoscope and aspirated into a trap connected to suction tubing. Usually 15-30 ml of fluid was instilled with each washing and about one-fourth to half of this volume was retrieved in the suction trap. Up to one-fourth of the instilled amount retrieved was considered successful. No studies, however, have established the ideal volume of fluid for optimum results. The bronchial washings were sent for AFB staining, AFB culture by BACTEC, and for cytology and cell count. Transbronchial lung biopsy was done with the biopsy forceps and sent for histopathological examination. In cases where an endobronchial growth was seen washing, brushing and biopsy were performed.

After the procedure, the patient was observed for development of pneumothorax, hemorrhage, infection and cardiac arrhythmias for 24-48 hours. The first sputum sample after bronchoscopy (post-bronchoscopic sputum) was collected and sent for analysis along with bronchial washings. Statistical analysis was done by McNemar test using SPSS 13.0.

## RESULTS

Bronchoscopy was performed on 75 patients. Characteristics of the patients are given in Table 1. The most common bronchoscopic finding was congestion with mild to moderate hyperemia with whitish plaques of variable size in between, observed in 53 (70.6%) patients. In 21 patients (28%), ulceration, erosion or granulation was seen. In all patients with cavitatory lesion the mucosa was ulcerated and swollen. In five patients ulcerative lesions were observed with extensive areas of pulmonary involvement radiographically. In 16 patients (21.3%) the segmental openings were narrowed and slightly deformed. Endobronchial growth was seen in three patients (4.0%). Transbronchial biopsy revealed caseating granuloma in 10 patients with acid fast bacilli in two patients only. Out of the 10 patients two had bronchial washings positive for acid fast bacilli. Non-caseating granulomas were observed in 19 patients suggestive of tuberculosis. This diagnosis was confirmed by pre-bronchoscopic sputum culture for AFB in 10, bronchial washings smear and culture for AFB in eight and post bronchoscopic sputum smear for AFB in one patient. Nonspecific chronic inflammatory changes were the findings in 27 patients and normal histology was observed in the remaining 15 patients.

**Table 1:** Patient characteristics

Patient characteristic	%
Sex	
Male	66.7
Female	33.3
Mean age (years)	
Total	43.20 ± 14.63
Male	45.20 ± 15.15
Female	39.20 ± 12.88
Mean duration of illness	2.2 months
Symptoms	
Cough	90.6
Expectoration	70.6
Fever	68.0
Constitutional symptoms	57.3
Dyspnea	26.6
Hemoptysis	16.0
Chest pain	14.6
Asymptomatic	2.7
Chest radiography	
Site of lesion	
Right	53.3
Left	37.3
Bilateral	9.3
Type of lesion	
Cavitatory	10.7
Non cavitatory	89.3

No serious complications were encountered during the study, except pneumothorax (less than 10%) in four patients and minimal hemoptysis (less than 10 ml) in 16 patients. No specific treatment was required to manage these complications. Three pre-bronchoscopic sputum smears were negative for acid fast bacilli in all the patients but culture was positive in 20 patients and this was the only diagnostic feature in 10 patients. Bronchial washings smear was positive in 21 patients. The culture of bronchial washings was positive for acid-fast bacilli in 39 patients. Patients with positive bronchial washing smear were positive on culture also. In 29 patients, smear or culture of bronchial washing alone contributed to the final diagnosis. The post-bronchoscopic sputum yielded acid fast bacilli in 11 patients [Table 2].

**Table 2:** Results of prebronchoscopic sputum culture and investigations performed on bronchoscopic specimens

Prebronchoscopic sputum-culture positive	Bronchial washing		Histopathology		Post bronchoscopy sputum	Total no. of patients
	Smear positive	Culture positive	Positive	Non-caseating granuloma	Smear positive	
4	-	-	-	4	2	4
-	-	13	-	-	3	13
-	-	-	8	-	-	8
-	-	-	-	1	1	1
-	14	14	-	8	-	14
5	5	5	-	3	-	5
-	2	2	2	-	-	2
3	-	3	-	-	3	3
8	-	-	-	3	-	8
-	-	2	-	-	2	2
20	21	39	10	19	11	60

Table 3 shows the relationship between prebronchoscopic sputum culture and bronchial washing culture in the 60 patients. McNemar test revealed that there was a significant difference in the results of the two tests (*P* is equal to 0.005). Thus, culture of bronchial washings gave a significantly more yield than pre-bronchoscopic sputum culture.

**Table 3:** Comparison of prebronchoscopic sputum culture and bronchial washings culture

Prebronchoscopic sputum culture	Bronchial washings culture		Total
	Positive	Negative	
Positive	8	12	20
Negative	31	9	40
Total	39	21	60

McNemar test, *P* = 0.005

Total yield of bronchoscopy in diagnosis of sputum smear negative pulmonary tuberculosis was 83.33% (50/60); bronchoscopy was the only diagnostic method in 66% cases (40/60) with bronchial washings being the only diagnostic method in 48.33%. Bronchial washings smear for AFB and histopathological evidence of caseating granuloma made immediate diagnosis possible in 48.33% (29/60) patients.

Bronchoscopy, along with various other contributory laboratory and radiological investigations, helped establish the diagnosis in nine more patients (three-adenocarcinoma, three-allergic bronchopulmonary aspergillosis and three-sarcoidosis). In the remaining six patients no specific diagnosis could be established either because investigations were incomplete and non-contributory or patients did not consent for further evaluation.

## DISCUSSION

In the earlier days of rigid bronchoscopy, patients with tuberculosis were seldom subjected to bronchoscopy for diagnostic purpose. With the advent of fibre-optic bronchoscopy, smear and culture for mycobacteria from the bronchial aspirate, bronchial brushing, bronchial washing, bronchoalveolar lavage fluid, postbronchoscopy sputum and biopsy material have all been used in various studies for diagnosing pulmonary tuberculosis.

In present study 70.6% of cases showed congestion with hyperemia of bronchial mucosa on bronchoscopy, 28% of patients had erosion, ulceration and granulation on bronchoscopy, all these were diagnosed as active pulmonary tuberculosis by demonstration of acid fast bacilli in bronchial washing smear or culture or postbronchoscopy sputum. While segmental narrowing was observed in 21.3% patients, four per cent of patients with intra-luminal growth were diagnosed as malignancy and later confirmed by the cytological examination of bronchial washing fluid as adenocarcinoma. Some cases had multiple findings on bronchoscopy.

Kulpati *et al*,[5] observed the coating of mucosa of involved segments with yellowish white secretions in almost all patients and also revealed mild to moderate hyperemia after bronchial wash. Segmental bronchus was narrowed in 20% patients, and ulceration was seen in 20% patients.

Purohit *et al*,[6] reported ulceration in 64% of patients; 60% had frothy secretion for the bronchus. A moderate hyperemia of bronchial mucosa was observed in all the patients.

Similar observations were made by Panda *et al*,[7] according to their study, 44% had normal bronchial mucosa, 21% had unhealthy mucosa with granulations, 35% had discharge of mucous from bronchus, five per cent had growth, three per cent had external compression and three per cent had bleeding from bronchus and some cases had multiple findings.

Flexible fibreoptic bronchoscopy provides material for early diagnosis e.g. bronchial washing for smear preparation and transbronchial biopsy for histopathological study. In our study bronchial washings smear was positive for acid fast bacilli in 35% patients; all of these were confirmed by positive culture. Danek and Bower[8] and Purohit *et al*.[6] demonstrated acid fast bacilli in 34% and 42% respectively where as in study by Kulpati *et al*,[5] 40% were positive. Wallace *et al*,[9] reported that transbronchial biopsy provided microscopic diagnosis in 30% and was the only diagnostic evidence in 26% of the patients. In the study conducted by Kulpati *et al*,[5] caseating granulomas were observed in four patients (20%) and were the only diagnostic feature in 15% of patients. In our study, caseating granulomas were observed in 16.7% and was the only diagnostic feature in 13.3% which is a lower diagnostic rate as compared to earlier studies.

In the study by Danek and Bower[8] granulomatous inflammation was included as diagnostic criteria for pulmonary tuberculosis and bacillary confirmation of diagnosis was present in 41% of their patients. The diagnosic yield of pulmonary tuberculosis increased to 55% in the study by Kulpati *et al*,[5] if non-caseating granulomas were also included. In our study the diagnostic rate was 61.6% if non caseating granuloma was also considered as diagnostic and this would be comparable to both the earlier studies.

Combining stained smear positivity of bronchial washings and histopathology, the diagnosis was established in 48.3% in our study while it was 48 and 50% in the studies conducted by Wallace *et al*.[9] and Kulpati *et al*.[5] respectively.

If granulomatous inflammation too is accepted as sufficient evidence for early diagnosis of tuberculosis, the diagnostic yield in our study will be 61.6% compared to 75% in the study by Danek and Brower.[8]

The results of stained smear examination of bronchial washing was confirmed by culture in 100% of cases in the present study, which is comparable to the studies by Kulpati *et al*.[5] (100%), Danek and Bower[8] (95%), Sarkar *et al*.[10] (87%), Uddenfeldt and Lundgren (83%).[11] In this study, bronchial washing-culture was positive for acid fast bacilli in 65% cases only, similar to that reported by Kulpati *et al*.[5] Kvale *et al*.[12] could grow acid fast bacilli only in one third of the patients of suspected tuberculosis. Kato *et al*.[13] reported that higher concentration of lidocaine had an inhibitory effect on mycobacterial growth. Though we did not culture the biopsy material, bacilli were grown in 20%, 60%, and 41% in studies of Wallace *et al*.,[9] Funahashi *et al*.[14] and Danek and Bower[8] but this was not the only diagnostic evidence in any of the studies and did not influence the diagnostic contribution of other methods. Kulpati *et al*.[5] reported that exclusively positive diagnostic results were provided by culture of prebronchoscopic sputum and bronchoscopic aspirate in five and 10% respectively while in our study these results were 16.7 and 16.7% respectively. Wallace *et al*.[9] and Danek and Bower[8] had reported 95% culture positivity of specimens obtained by flexible fibreoptic bronchoscopy and therefore negative culture provided strong evidence against tuberculosis.

In our study, the culture positivity of the specimen obtained by flexible fibreoptic bronchoscope was 65%. This disparity may be because transbronchial biopsy and post bronchoscopy sputum culture was not done in our study. Post-bronchoscopy sputum studies provide collaborative evidence in sputum/smear-negative pulmonary tuberculosis. In various previous studies flexible fibreoptic bronchoscopy in combination with transbronchial lung biopsy provided early diagnosis in 60% to 85% of smear negative pulmonary tuberculosis. In our study flexible fibreoptic bronchoscopy provided the diagnosis in 83.33% of patients which is similar to the previous studies. Table 4 compares the results of our study with that of various previous studies.

**Table 4:** Comparison of the present study with previous studies in literature

Author, year of publication, country	Yield for tuberculosis % (No. of cases)	Immediate diagnosis % (No. of cases)	Exclusive diagnosis % (No. of cases)
Danek **et al**.,[8] 1979, USA	95 (39/41)	34 (14/41)	46 (19/41)
Sarkar *et al*.,[10] 1980, India	87 (26/30)	73 (22/30)	NS
Wallace *et al*.,[9] 1981, USA	65 (15/23)	48 (11/23)	22 (5/23)
Uddenfeldt *et al*.,[11] 1981, Sweden	83 (25/30)	NS	37 (11/30)
So *et al*.,[15] 1982, Hong Kong	94 (61/65)	65 (42/65)	NS
Stenson *et al*.,[16] 1983, USA	66 (8/12)	42 (5/12)	NS
Russel *et al*.,[17] 1986, USA	100 (25/25)	12 (3/25)	40 (10/25)
Kulpati *et al*., 1986[5] , India	60 (20/33)	40	NS
Palenque *et al*.,[18] 1987, Spain	100 (50/50)	34 (17/50)	NS
Wongthim *et al*.,[19] 1989, Thailand	76 (54/71)	75 (53/71)	11 (8/71)
Khoo *et al*.,[20] 1989, UK	26 (9/35)	9 (3/35)	17 (6/35)
Zainudin *et al*.,[21] 1991, Malaysia	100 (33/33)	55 (18/33)	NS
Fujii *et al*.,[22] 1992, Japan	91 (29/32)	44 (14/32)	34 (11/32)
Present study (2007)	80 (60/75)	48.3	66

Our study suggests that fibre-optic bronchoscopy can provide excellent material for diagnosis of suspected cases of pulmonary tuberculosis when smears of expectorated sputum do not reveal mycobacteria. Fibre- optic bronchoscopy combined with transbronchial lung biopsy helps in early diagnosis of smear negative pulmonary tuberculosis in a significant number of cases.
